# ADSP Whole Genome Sequencing (WGS) Release 5 data update from Genome Center for Alzheimer’s Disease

**DOI:** 10.1002/alz.087495

**Published:** 2025-01-03

**Authors:** Luke Carter, Yuk Yee Leung, Wan‐Ping Lee, Amanda B Kuzma, Prabhakaran Gangadharan, Heather Issen Nicaretta, Liming Qu, Youli Ren, Otto Valladares, Yi Zhao, Taha Iqbal, Michael A. Schmidt, Pedro R. Mena, The Alzheimer’s Disease Sequencing Project (ADSP), Clifton L. Dalgard, Brian W. Kunkle, William S. Bush, Eden R. Martin, Adam C. Naj, Johnathan L Haines, Margaret A. A Pericak‐Vance, Li‐San Wang, Gerald D. Schellenberg, Alzheimer’s Disease Sequencing Project

**Affiliations:** ^1^ Penn Neurodegeneration Genomics Center, Department of Pathology and Laboratory Medicine, Perelman School of Medicine, University of Pennsylvania, Philadelphia, PA USA; ^2^ Department of Biostatistics, Epidemiology, and Informatics, Perelman School of Medicine, University of Pennsylvania, Philadelphia, PA USA; ^3^ John P. Hussman Institute for Human Genomics, University of Miami Miller School of Medicine, Miami, FL USA; ^4^ Department of Anatomy, Physiology, and Genetics, Uniformed Services University of the Health Sciences, Bethesda, MD USA; ^5^ Cleveland Institute for Computational Biology, Department of Population and Quantitative Health Sciences, Case Western Reserve University, Cleveland, OH USA; ^6^ University of Pennsylvania, Philadelphia, PA USA

## Abstract

**Background:**

The Genome Center for Alzheimer’s Disease (GCAD) coordinates the integration and meta‐analysis of all available Alzheimer’s disease (AD) relevant whole genome sequencing (WGS) data to facilitate the goal of identifying AD risk or protective genetic variants and eventual therapeutic targets. The WGS datasets are generated via the collaboration of scientists from the Alzheimer’s Disease Sequencing Project (ADSP) and GCAD. To minimize data heterogeneity introduced by different sequencing protocols and machines, GCAD processes all samples using identical pipelines.

**Methods:**

The raw sequencing data are first mapped to GRCh38/hg38 and variants (SNVs and indels) are called using GATK. Additionally, compact VCF and GDS formatted files are generated to facilitate researchers who want to use smaller pVCFs. SNVs and indels are annotated using the ADSP annotation pipeline. Lastly, structural variants (SV) are called using Smoove and Manta and joint genotyped using GraphTyper2.

**Results:**

The dataset (ADSP Release 5, R5, 2024) includes ∼60,000 genomes from >50 diverse cohorts with 4 major ancestries: 47% Non‐Hispanic White, 29% Hispanic or Latino, 16% Black or African American and 8% Asian. Data are deeply sequenced (average genome coverage: >30x). CRAMs, gVCFs from GATK, and SV VCFs of a subset of the R5 samples (n = 36,361) were deposited into NIAGADS Data Sharing Service (DSS) (https://dss.niagads.org/) for public distribution in 2022, and similarly, the new samples in R5 will be released after the joint call is complete. In addition, joint‐genotype VCFs on SNVs, indels, and SVs will be available. These will undergo full quality control and annotation process.

**Conclusion:**

The ADSP and GCAD generate high quality genotype and SV calls. Currently the project is processing ∼60,000 WGS samples sequenced primarily through the ADSP Follow‐Up Study, which will contain a more ancestrally diverse set of populations. We anticipate this 2024 release will continue to benefit the research community studying AD genetics.

